# Rapid detection of allelic losses in brain tumours using microsatellite repeat markers and high-performance liquid chromatography

**DOI:** 10.1038/sj.bjc.6601025

**Published:** 2003-06-10

**Authors:** O B Chernova, G H Barnett, J K Cowell

**Affiliations:** 1 Department of Neurosurgery, Cleveland Clinic Foundation, 9500 Euclid Avenue, Cleveland, OH 44195, USA; 2 Department of Cancer Genetics, Roswell Park Cancer Institute, Elm and Carlton Streets, Buffalo, NY 14263, USA

**Keywords:** brain tumours, denaturing HPLC, loss of heterozygosity

## Abstract

High-performance liquid chromatography (HPLC) is a recently introduced high-capacity automated method for detecting unknown mutations (denaturing HPLC) or for sizing DNA fragments under nondenaturing conditions. We have adapted the HPLC method for detection of loss of heterozygosity (LOH) and used glial tumours as a model to evaluate its sensitivity and specificity in comparison to conventional denaturing polyacrylamide gel electrophoresis. A total of 20 oligodendrogliomas (grades II and III), and five astrocytic tumours (grades III and IV) were analysed using 14 polymorphic microsatellite markers mapping to regions on chromosomes 1p, 19q, and 10q using both DNA-HPLC and denaturing gel electrophoresis. This study demonstrated complete concordance between both methods. However, unlike gel electrophoresis, HPLC is automated, does not require post-PCR processing, and does not require hazardous radioactive or expensive fluorescent labelling. Our data suggest that HPLC is a reliable and sensitive method for detection of allelic losses in tumour samples and it is a favourable alternative for high-sensitivity LOH detection in both research and diagnostic environments.

Molecular genetic analysis of tumours frequently demonstrates that regions of chromosomes that were heterozygous in constitutional cells from patients become homozygous in the tumour (Pal *et al*, 1990; Wadey *et al*, 1990; Grundy *et al*, 1998). This loss of heterozygosity (LOH) has frequently been shown to result in the loss of the chromosome region that contains the normal allele of a gene critical for tumourigenesis. This mechanism results in the ‘exposure’ of recessive mutations in the homologous gene on the remaining chromosome (Cavenee *et al*, 1983; Solomon *et al*, 1987; Xu *et al*, 1992; Kelsell *et al*, 1993). These observations have been so consistent that LOH analysis has now become a primary tool to identify regions of chromosomes thought to harbour genes responsible for the development and progression of a wide variety of tumours. Recently, LOH has also been used as a diagnostic tool to predict whether anaplastic oligodendrogliomas (AO) will be sensitive or resistant to chemotherapy (Cairncross *et al*, 1998; Smith *et al*, 2000). In these studies, AO showing LOH for the short arm of chromosome 1 (1p) invariably demonstrate a positive response to chemotherapy, whereas patients who have retained heterozygosity in their tumours were resistant to chemotherapy. This particular LOH assay provides a very important test for the clinical management of patients with AO. Clearly, however, it is essential to establish the LOH status of these tumours quickly and accurately in order to be able to customise therapy and begin it in a reasonable time following biopsy.

Currently, the main method used to establish the LOH status of tumours involves using microsatellite repeat marker analysis from DNA isolated either from fresh tissue or paraffin-embedded sections. These techniques typically require radioactive polymerase chain reaction (PCR) amplification, acrylamide gel analysis and autoradiography of the region containing the microsatellite repeat. Depending on the particular marker used, the results are sometimes difficult to interpret because of the ‘stutter’ bands that can accompany the alleles (especially when alleles are similar in size), which results from slippage of the template during PCR amplification. This whole procedure can also be time consuming. Despite these limitations, this technology is still the most frequently used to define regions of LOH.

Recently, it has become possible to eliminate the use of gels, radioactivity, and autoradiography by adapting DNA-high-performance liquid chromatography (DNA-HPLC) approaches to the analysis of DNA fragments. The Transgenomics WAVE system, for example, provides an automated way to perform heteroduplex analysis that can detect mismatches between paired samples. This procedure has found more extensive use as a quick and reliable way of identifying mutations in PCR products amplified from specific genes (O'Donovan *et al*, 1998; Arnold *et al*, 1999; Choy *et al*, 1999; Gross *et al*, 1999). However, DNA samples from cells that are heterozygous for microsatellite markers contain different sized alleles, and so the question, therefore, is whether allelic loss can be identified using HPLC in order to streamline the identification of LOH in tumour samples. To investigate this, we have analysed polymorphic microsatellite markers from the short arm of chromosome 1 (1p) and the long arm of chromosome 19 (19q) in order to determine whether it is possible to adapt the HPLC analysis to the problem of predicting chemosensitivity in AO. Here we report that, with the appropriate selection of microsatellites and the correct conditions of the temperature gradient, LOH can clearly be established efficiently and far more quickly than using conventional approaches.

## MATERIALS AND METHODS

### Tumour samples and DNA isolation

Brain tumour tissues and peripheral blood samples were obtained at the time of surgery from patients attending the Cleveland Clinic Foundation Department of Neurosurgery. Representative tumour samples were snap frozen and stored at −70°C. Blood lymphocytes were purified using Ficoll gradients from 3–5 ml of heparin-treated blood and stored at −70°C in 10% DMSO. DNA was isolated from frozen or fresh lymphocytes or tumour tissues using DNA-Easy reagents (Invitrogen) according to the manufacturer's instructions.

### Microsatellite markers

A total of 14 genetically mapped dinucleotide and tetranucleotide microsatellite markers were used for LOH analysis. The genetic location, primer sequences, and the size of the product of each marker were obtained from the Genome Database (http://gdbwww.gdb.org/). Primers were synthesised commercially (Research Genetics, Inc., AL, USA; Genosys Biotechnologies Inc., TX, USA).

### Microsatellite HPLC analysis

Polymerase chain reaction was performed in a volume of 25 *μ*l containing 1 × *Taq* buffer, 100 ng of genomic DNA, 0.2 *μ*M each primer, 1.7 mM MgCl_2_, 200 *μ*M each dNTP. 1.0 U of *Taq* (Gibco, BRL) and 0.2 U of *Pfu* (Promega) polymerases. Amplification was performed in a PTC-100 thermocycler (MJ Research Inc., MA, USA). Cycling was performed with an initial denaturation step of 3 min at 94°C followed by 30 cycles of 94°C for 45 s, 56°C for 30 s, and 72°C for 50 s and then an extension step at 72°C for 3 min. High-performance liquid chromatography analysis was carried out on the automated WAVE™ DNA Fragment Analysis System (Transgenomics, Inc., CA, USA). Polymerase chain reaction products were separated using a flow rate of 0.9 ml min^−1^ over a period of 5–10 min through a linear acetonitrile gradient consisted of a mixture of 0.1 M triethylamine acetate (pH 7.0) without (buffer A) or with (buffer B) 25% acetonitrile. The values for the buffer gradient were calculated by the WaveMaker software according to the size of the amplicon. The temperature of separation column was fixed at 50°C, which is the temperature recommended for size separation by the manufacturer.

### Microsatellite electrophoresis analysis

Polymerase chain reaction amplification of the markers was performed in 25 *μ*l containing 50 ng of genomic DNA, 0.2 *μ*M of each primer (one of them end-labelled with *γ*-^32^P), 1.7 mM MgCl_2_, 200 *μ*M each dNTP, 1 × amplification buffer, and 0.5 U of *TaI* polymerase (Gibco, BRL). Cycling was performed as described above. The products of the PCR reaction were mixed with an equal volume of formamide loading buffer (95% formamide, 20 mM EDTA, 0.05% bromophenol blue, 0.05% xylene cyanol), denatured at 95°C for 5 min, and cooled on ice. A volume of 3 *μ*l of each sample was loaded on a 6% polyacrylamide gel containing 8 M urea and electrophoresed for 2.5 h at 65 W. Gels were dried and exposed to Kodak XAR film for 4–24 h.

## RESULTS AND DISCUSSION

For routine clinical diagnostic use, it is necessary for test procedures to be quick and accurate and relatively inexpensive. We set out to establish, therefore, using brain tumours as an example whether ion-pair(IP)-reverse phase (RP) HPLC could be used for detection of LOH in a variety of brain tumours. Loss of heterozygosity analysis was performed using paired DNA samples from tumour and blood from 25 patients with glial tumours. Polymerase chain reaction products were generated from microsatellite markers from chromosomes 1p, 19q, and 10q (Table 1

**Table 1:** Loss of heterozygosity (LOH) frequencies of the 1p, 19q, and 10q markers in gliomas

**Marker**	**Chromosome**	**Repeat type, bp**	**ODG II–III**	**GBM**	**AA**	**MOA**
D1S552	1p36	4	7^a^/14^b^	1/3	0/1	1/3
D1S1612	1p36	4	6/11	1/3	0/2	0/2
D1S468	1p36	2	4/8	1/2	ND	0/2
D1S551	1p31	4	8/9	1/4	0/1	1/4
D1S430	1p31	2	4/9	0/2	0/1	1/2
D19S254	19q13.4	4	6/9	1/2	0/3	0/4
D19S572	19q13.4	2	2/3	2/3	ND	ND
D10S587	10q26	2	0/5	3/4	ND	ND
D10S209	10q25	2	2/6	2/3	ND	ND
Tumours analysed			18	4	3	5

**Figure 1:**
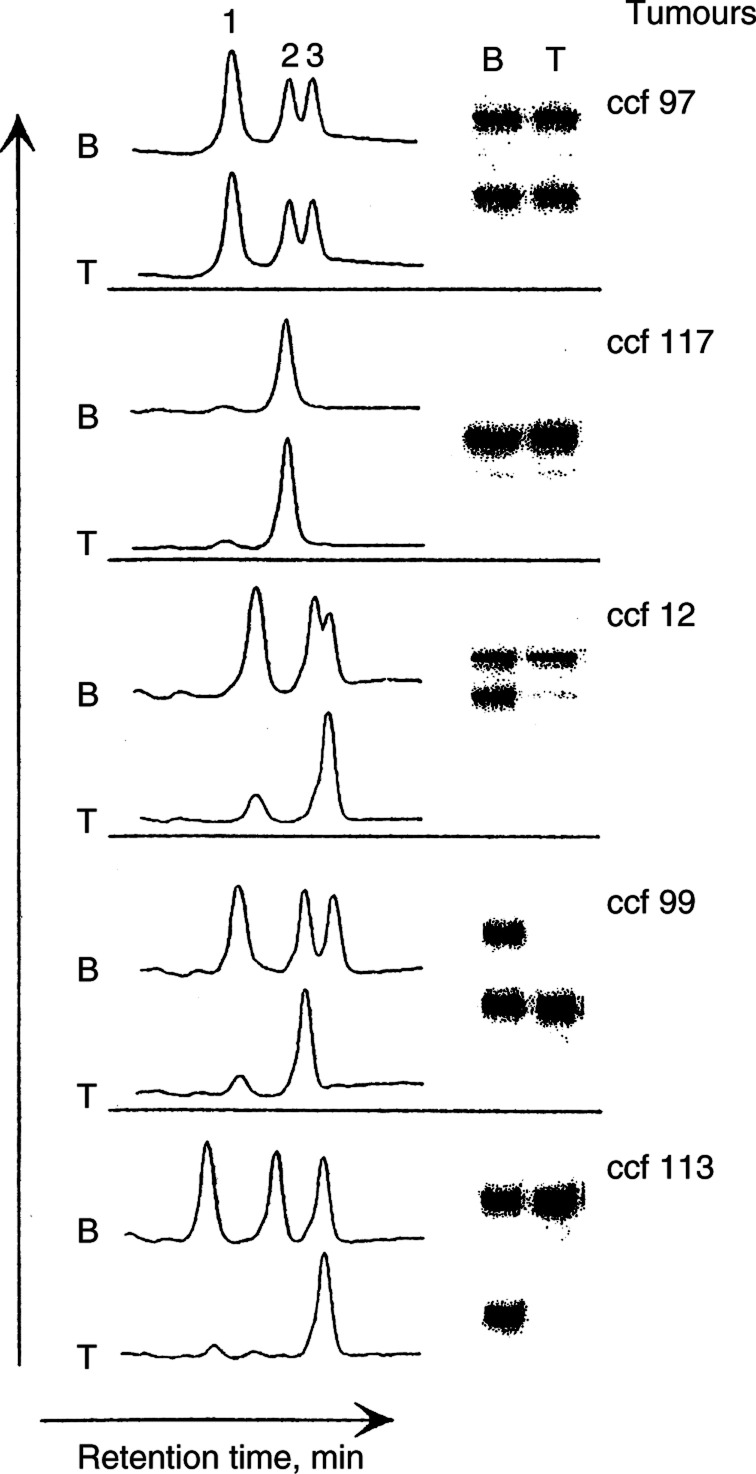
Comparison of LOH analysis using DNA-HPLC and denaturing polyacrylamide gel electrophoresis. Examples from five tumours using marker D1S551 are shown. DNA from peripheral blood (B) or tumour (T) were analysed by both methods. Electrophoresis results are shown on the right. The relevant portions of the corresponding HPLC elution profiles are shown on the left in each example. Peaks 2 and 3 represent the two allelic homoduplexes of D1S551 in normal DNA from individuals heterozygous for the marker. Peak 1 represents a heteroduplex with shorter retention time. Tumour ccf 117 is constitutionally homozygous and shows only one peak. Tumour ccf 12 is heterozygous, but the two alleles differ in size by only four base pairs and so the two allelic peaks in this case do not resolve as well as in the other examples shown. In the three tumours showing LOH, the presence of a single peak in the tumour indicating the retained allele together with the loss of the heteroduplex peak can be clearly seen. The elution time (min) is represented on the *x*-axis, and the ultraviolet absorbance at 260 nm is represented on the *y*-axis (in *μ*V).

**Figure 2:**
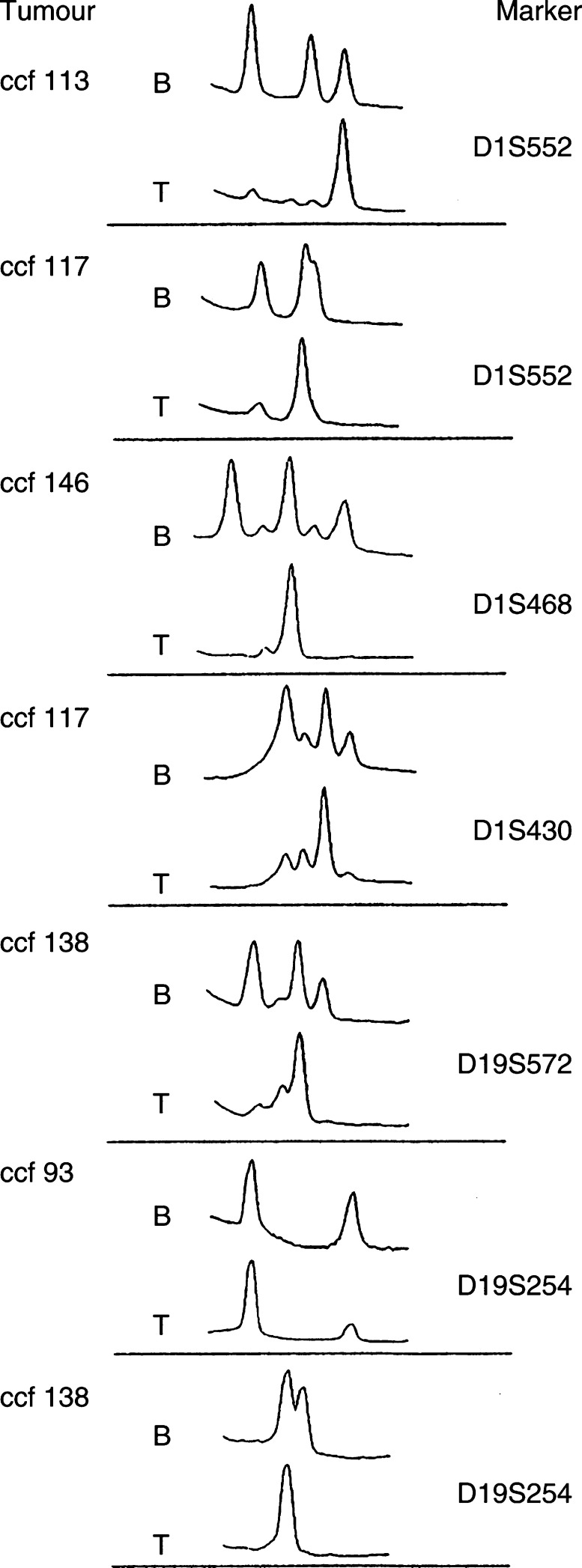
High-performance liquid chromatography detection of LOH in glial tumours. Representative chromatograms from seven different tumour (T)/normal (B) comparisons are shown. Many of the markers show the clear results described for marker D1S551. Two markers, D1S430 and D19S572, show evidence of stutter bands in the PCR products as seen by the minor peaks flanking the main peak. Even despite the extra ‘noise’ seen in the chromatogram, it is still relatively easy to identify LOH in these cases by the presence of only a single main peak.

ODG=oligodendroglioma grades II and III; GBM=glioblastoma multiforme: AA=anaplastic astrocytoma; MOA=mixed oligoastrocytoma.

a Number of samples with LOH.

b Number of informative samples.

) and then subjected to IP-RP HPLC under nondenaturing conditions (50°C). The elution profiles for the PCR products from each of the microsatellites were compared to the gel patterns obtained for the same markers using standard denaturing polyacrylamide gel electrophoresis of ^32^P-labelled PCR fragments. Figure 1

shows examples of the elution profiles (chromatograms) and corresponding gel patterns of PCR fragments from a series of tumours with or without LOH for the D1S551 marker. Two allelic microsatellite fragments (heterozygous) were detected in the constitutional DNA of patient ccf 97 after separation of ^32^P-labelled PCR products on denaturing acrylamide gel. High-performance liquid chromatography analysis of the same DNA sample under nondenaturing conditions shows three peaks on the elution profile. Peaks 2 and 3 correspond to the homoduplexes derived from the two different sized alleles. Peak 1 represents the heteroduplex formed between the two different sized PCR products during the last cycle of the PCR reaction. Because of the mismatches in the heteroduplex, these molecules have a reduced retention time on the column and so are eluted ahead of the homoduplexes. In this example, ccf 97, the profiles are identical in both tumour and normal DNA indicating that there has been no LOH. The example shown for ccf 117 reveals that using acrylamide gels the patient is homozygous for the D1S551 marker (Figure 1) and a single peak is seen on the HPLC chromatogram. This patient is referred to as ‘not informative’ since it cannot be established whether an allele has been lost or not. Tumours where LOH can be detected were derived from patients ccf 12, ccf 99, and ccf 113. In all of these cases, loss of one allele is indicated by a significant reduction in the size of one of the homoduplexes, which is accompanied by a reduction or elimination of the heteroduplex signal (Figure 1). It is likely that the presence of a residual band in the heteroduplex, for example, in ccf 12, is due to low-level contamination by normal cells in the original tumour sample. As with all LOH approaches, the percentage of contaminating normal cells will affect the appearance of the elution profile, although in our studies this has not been a significant problem. It appears that reduction/elimination of the heteroduplex signal is a very accurate and sensitive indicator of LOH, especially in cases (e.g. ccf 12) where, because of the small difference in allele sizes, both homoduplex peaks are poorly separated (Figure 1). It is clear from these studies that there is excellent concordance between HPLC and denaturing gel analysis. In the initial studies, we used the microsatellite marker D1S551 that was known from our previous analysis to generate discrete PCR products for each of the alleles. This is not always the case with microsatellite markers since, because of the highly repetitive nature of the repeat they contain, template slippage often occurs, which results in ‘stutter’ bands on either side of the main PCR product. To test the influence this would have on the HPLC elution profile, we analysed a number of different microsatellite markers (Table 1) that mapped to chromosomes 1p, 19q, and 10q. These markers are spread across regions that frequently show LOH in glial tumours. It can be seen from the representative chromatograms shown in Figure 2

that a variety of marker-specific elution profiles are generated. Some markers, for example, D1S552 (Figure 2), have heterozygous elution profiles that showed the typical three peaks similar to D1S551, and so LOH is easily seen. Other profiles, such as the ones for D1S468 and D1S430, show the stutter peaks but with considerably reduced intensity and so these can effectively be ignored. In fact, the shape of the chromatogram depends on three factors: (1) the size difference between two allelic variants (heteroduplex formation is suppressed when the size of the two alleles are very different), (2) primary/secondary structure of the PCR fragment, and (3) the abundance of the stutter PCR products. Despite the complex patterns of elution profiles, loss of one allele in heterozygous samples is always easily detectable, since the retained allele produces only a single peak. Despite the generally consistent observation that loss of the heteroduplex peak indicates LOH, for some microsaellite markers this was not the case. An example is shown in Figure 2 for the D19S254 marker. In this case, a heteroduplex is apparently not formed, or coelutes with one of the homoduplex peaks. In this case, however, LOH can still easily be detected based on the loss of one of the peaks in both ccf 93 and ccf 138. This rare elution profile underscores the need to select markers for LOH studies carefully and to determine their elution profiles empirically.

Importantly, we have been able to show that the elution profile for any given marker is highly reproducible in parallel HPLC runs. Furthermore, tumour samples that have retained both chromosomes demonstrate elution profiles almost identical to the corresponding blood DNAs. The other important point is that any change in the elution profile in the tumour samples invariably indicated loss of one allele. Clearly, the choice of the marker to be used is important since some microsatllites, regardless of which technique is used, produce band profiles which cannot be interpreted. It is usually necessary, therefore, to identity several microsatellite markers from the region of interest if possible, and then select the ones that give the clearest results for routine testing. In addition to the nine markers listed in Table 1, we also analysed 12 more markers and found that some of them (D1S224, D1S216, D10S185, D19S216, and D19601) produce easily readable elution profiles (data not shown) and can also be used for LOH analysis.

Our original reason for adapting the WAVE technology to the detection of LOH using microsatellite markers was to investigate its applicability to the analysis of brain tumours to determine their chemosensitivity. We therefore selected nine markers from chromosomes 1p, 19q, and 10q and analysed LOH status for 30 pairs of blood and glial tumour DNA using the WAVE system. In all, 82% (125 of 152) of these analyses demonstrated constitutional heterozygosity and so could be used for LOH detection. Table 1 summarises the informative cases, divided into morphological subtype, showing loss of the respective 1p, 19q, and 10q regions. In our analysis we focused mainly on oligodendroglial tumours, although other types of glial tumours were also included. Oligodendrogliomas grades II and III showed loss of 1p in 10 of 18 (55%) tumours, and loss of 19q in 7 of 12 (58%) informative cases. All tumours with 19q allelic loss also demonstrated LOH on 1p. These data are in agreement with recent reports that demonstrated the loss of 1p and 19q in 40–80% of oligodendroglial tumours (Ransom *et al*, 1992; Reifenberger *et al*, 1994; Kraus *et al*, 1995; Zhu *et al*, 1998). Since this is a prospective study, details about response to therapy and prognosis are not available at this time. Unlike astrocytic tumours (Fults and Pedone, 1993; Karlbom *et al*, 1993; Rasheed *et al*, 1995; Kimmelman *et al*, 1996; Ichimura *et al*, 1998), which demonstrate frequent loss of 10q (up to 80% in glioblastoma multiforme (GBM)), oligodendrogliomas have been less completely characterised (Maier *et al*, 1997; Rasheed *et al*, 1999). Although we have only analysed a few tumours, since the focus of this study was to compare WAVE analysis with gel analysis, we nonetheless found loss of 10q in two of five oligodendrogliomas and two of two 2 GBMs. 42% of the microsatellite loci analysed in this study were also analysed using standard polyacrylamide gel electrophoresis and, in all cases, the results were completely concordant with those from the HPLC.

The analysis of these brain tumours revealed an important detail that reinforces the need to carefully select the microsatellites to be used in WAVE-LOH. From the results in Table 1, several of the markers, for example, D19S572 and D10S587, showed relatively few heterozygotes in our small series. All of these markers represent dinucleotide repeats that supposedly have a heterozygosity frequency of over 70% in the general population.

Although it is possible that the decreased frequency of observed heterozygotes is a consequence of sampling bias in this small population, it is also possible that for some markers where the size differences between alleles are small, HPLC may not be able to resolve the individual alleles. The frequency of heterozygotes for the tetranucleotide repeats markers, however, were generally close to those expected. Clearly, if the analysis permits, it would be preferable to use this type of marker in this application of LOH analysis.

In summary, this study demonstrates that HPLC is a reliable and sensitive alternative for the detection of allelic losses in tumour samples. It is usually necessary to screen several microsatellite markers from the same interval if possible since some, despite which chromosome region is selected, are difficult to interpret regardless of the method used. Importantly, unlike denaturing gel electrophoresis, HPLC analysis is automated, does not require post-PCR processing, and does not require radioactive or expensive fluorescence labelling. This technique is also less prone to artefacts produced by technical problems with creating and running gels, such as edge effects or incomplete polymerisation, which, might further complicate the analysis of the results. High-performance liquid chromatography, therefore, is a very favourable alternative for rapid, high-throughput LOH detection in both research and diagnostic environments.
